# Effects of Some Olive Fruits-Derived Products on Oxidative Stress and Cardiovascular Biomarkers on Experimental Diabetes Mellitus

**DOI:** 10.3390/antiox13091127

**Published:** 2024-09-18

**Authors:** José Pedro De La Cruz, Laura Iserte-Terrer, María Dolores Rodríguez-Pérez, Laura Ortega-Hombrados, Ana María Sánchez-Tévar, María Monsalud Arrebola-Ramírez, María África Fernández-Prior, Cristina Verdugo-Cabello, Juan Antonio Espejo-Calvo, José Antonio González-Correa

**Affiliations:** 1 Departamento de Farmacología, Instituto de Investigación Biomédica de Málaga y Plataforma en Nanomedicina—IBIMA Plataforma BIONAND, Facultad de Medicina, Universidad de Málaga, 29590 Málaga, Spain; jpcruz@uma.es (J.P.D.L.C.); laura.iserte@uma.es (L.I.-T.); hombrados@uma.es (L.O.-H.); amstevar@uma.es (A.M.S.-T.); cristinaverdugocabello@uma.es (C.V.-C.); correa@uma.es (J.A.G.-C.); 2 UGC Laboratorio Clínico, Hospital de la Axarquía, AGSEMA, 29740 Málaga, Spain; mariam.arrebola.sspa@juntadeandalucia.es; 3 Consejo Superior de Investigaciones Científicas (CSIC), Instituto de la Grasa, 41013 Sevilla, Spain; mafprior@ig.csic.es; 4 Tecnofood I+D+i Soluciones S.L., Instituto para la Calidad y Seguridad Alimentaria (ICSA), 18320 Granada, Spain; jaespejo@hotmail.com

**Keywords:** olive oil, diabetes mellitus, vascular complications, oxidative stress

## Abstract

The aim of this study is to assess the possible effect of olive seed oil (OSO) and destoned and dehydrated olive oil (DDOO), in comparison with extra-virgin olive oil (EVOO), on some cardiovascular biomarkers in an experimental model of diabetes mellitus. Diabetic animals showed evident alterations in biomarkers involved in the evolution of diabetic vasculopathy, marked by increases in biomarkers that favor vascular damage, which was between 1.5 and five times as many as those in non-diabetic animals, and a smaller number of biomarkers that protect against such damage (25–75% less than in healthy controls) was observed. The three oils administered decreased the concentration of biomarkers of vascular damage (35–45% in the serum lipid profile, 15–40% in early biomarkers of vascular inflammation and 20–60% in platelet aggregation and in thromboxane/prostacyclin imbalance). The greatest effect was by the antioxidant, both in the inhibition of lipid peroxidation and in the increase of glutathione. DDOO showed a significantly greater effect on oxidative stress and on thromboxane/prostacyclin imbalance than those shown by OSO and EVOO. This greater effect may possibly be explained by its higher triterpenoid content (913 mg/kg, compared to 113 mg/kg in OSO and 75 mg/kg in EVOO). We conclude, in the light of the results of this study, that these oils meet two basic conditions: they could improve the yield of the olive industry, and they equal, and may even increase, the beneficial effects of EVOO on cardiovascular disease.

## 1. Introduction

Olive oil is the main product obtained from olives, and its production and marketing are the mainstay of the olive sector. The production and quality of olive oil have improved in recent decades with the development of whole-olive—including the stone—milling procedures in continuous centrifuges (two and three phases) that have replaced the traditional pressing process [1].

The main environmental and economic problem is the enormous number of by-products that originate in the production of oil. Approximately 5 kg of olives generate 1 L of oil and more than 4 kg of by-products, which means there is millions of tons of waste that is difficult to store and costly to treat to reduce their toxicity or to process [2].

Although the agronomic interest in reusing waste not used in the production of extra-virgin olive oil (EVOO) and increasing the efficiency of the process of obtaining EVOO is important, it is necessary to take into account that this oil has a number of proven health benefits, so if the processes of extraction and handling of olives are to be modified, it is mandatory to verify that the final result retains these beneficial properties [3,4].

It has been widely demonstrated that the incorporation of EVOO to the diet, mainly the Mediterranean type, is a key element in understanding the benefits of this type of diet [3,4]. Both experimentally and in humans, EVOO has been shown to prevent the onset of cardiovascular and neurodegenerative events, some types of cancers, and even metabolic syndromes. These effects of EVOO have been attributed mainly to the content of polyphenolic compounds, mainly alcoholic (hydroxytyrosol, tyrosol and 3,4-dihydroxyphenyl-glycol) and triterpenoids (oleacein, maslinic and ursolic acid), among others [5,6]. Many studies have been carried out on these compounds and their associations, postulating the possibility of synergy between them [7,8,9].

Likewise, the effect of these compounds is related to their antioxidative and anti-inflammatory effects, deriving from these the other of the effects demonstrated in various aspects of biochemistry and cellular functionalism [5].

In recent years, we have studied the effect of these polyphenols and their association in various aspects of the complications of diabetes mellitus as one of the main cardiovascular risk factors [8,10,11,12,13,14], so we set out to evaluate, in this experimental model, two olive derivatives, in comparison with EVOO: olive seed oil and olive oil from destoned and dehydrated olives.

The aim of this study is to assess the possible effect of olive seed oil and destoned and dehydrated olive oil, in comparison with extra-virgin olive oil, on some cardiovascular biomarkers in an experimental model of diabetes mellitus. Concomitantly, this study aims to assess whether the composition of these oils could explain these possible differences.

## 2. Materials and Methods

### 2.1. Analytical Reagents

Most of the determinations were performed using commercial kits, some colorimetric and other enzyme-linked immunosorbent assay (ELISA). The 11-dehydrothromboxane B_2_, 3-nitrotyrosine and 6-keto-prostaglandin F_1α_ (6-keto-PGF_1α_) enzyme immunoassay kits are from Cayman Chemical (Ann Arbor, MI, USA). The 8-isoprostane enzyme immunoassay and total antioxidant capacity colorimetric kits are from Cell Biolabs, Inc. (San Diego, CA, USA). VCAM-1, myeloperoxidase (MPOx) and oxidized low-density lipoprotein (oxLDL) enzyme immunoassay kits are from Abyntek Biopharma, S.L. (Bilbao, Spain). Glutathione concentration and glutathione peroxidase activity kits and 8-hydroxy 2-deoxyguanosine enzyme immunoassay kits are from Abcam plc (Cambridge, CB2 0AX, UK). Collagen was obtained from Menarini Diagnóstica (Barcelona, Spain). All other reagents are from Merck Life Science S.L.U. (Madrid, Spain).

### 2.2. Olive Fruit Derived Products

**Figure 1:**
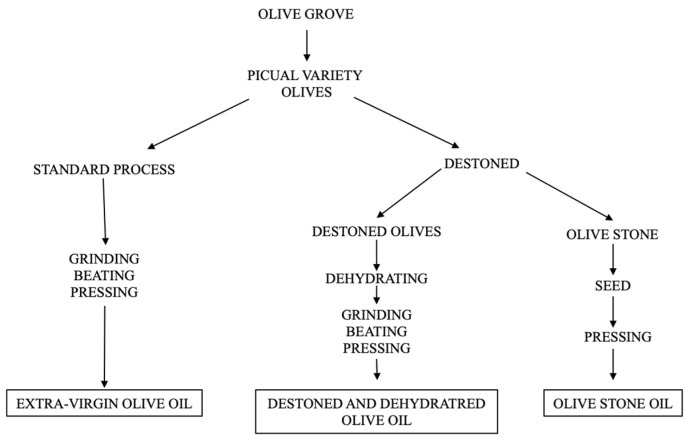
Basic outline of the procedures for obtaining the three types of oils.

Three types of oils were used; all of them are from the Picual variety olive, obtained and processed in the company Emilio Vallejo S.A. (Jaén, Spain). The procedure followed from the harvesting of the Picual variety olives is as follows (Figure 1):

- Extra-virgin olive oil (EVOO)

It was obtained from whole Picual-variety olives using the two-stage cold extraction system, which is the most used method in Spain. The process includes separation of leaves and sticks, milling, beating and pressing of the paste obtained, washing with water, decanting and obtaining the final EVOO. This procedure was carried out in the company Emilio Vallejo S.A. (Jaén, Spain).

- Olive seed oil (OSO)

It was obtained by mechanical extraction by cold pressing after the fragmentation and drying of the olive pit seeds and the drying phase, as described in patent 2389816 [15]. Briefly, in the first stage, whole olives are sorted by size; in the second stage, they are taken to a stone splitter, which splits the stone without damaging the pit. In the third stage, they are taken to a sorting separator, which separates pips or kernels from the shell; in the fourth stage, they are passed through an artificial vision sorter, which discards all the defective pips. In the fifth stage, the seeds are subjected to gentle drying to leave them with the necessary moisture for further processing; in the sixth stage, they are subjected to cold pressing in a continuous press. In the seventh stage, the oil obtained is filtered.

This procedure was carried out in the company Acer Camprestres S.L. (Jaén, Spain).

- Destoned and dehydrated olive oil (DDOO)

To obtain this oil, the olives are cleaned, stoned and then dehydrated at a temperature not exceeding 40 °C. Finally, the dehydrated pulp is continuously centrifuged to obtain the final oil. The procedure is described by Olmo-García et al. [16]. The process comprises the following steps: (a) obtaining olive pulp; (b) dehydration of the olive pulp, resulting in dehydrated olive pulp; (c) milling of the dehydrated olive pulp, resulting in dry dehydrated olive powder; and (d) obtaining olive oil from the dry dehydrated olive powder.

This procedure was carried out in the company Acer Camprestres S.L. (Jaén, Spain).

Table 1 shows the composition of the three types of oils administered to the experimental animals. The fatty acid composition of the oil was analyzed by simultaneous oil extraction and fatty acid methylation of the extracted oils. A gas–liquid chromatograph (GLC) PerkinElmer Clarus 600 GC (PerkinElmer Inc., Waltham, MA, USA) was used. The GLC was equipped with a BPX70 (30 m × 0.25 mm internal diameter × 0.25 µm film thickness) capillary column (SGE Analytical Science Pty, Ltd., Ringwood, Australia). A split injector and a flame ionization detector were maintained at 300 °C, using hydrogen as a carrier gas (0.8 mL/min). The determination of aliphatic alcohols, sterols and triterpenic dialcohols (erythrodiol and uvaol) was carried out in an Agilent 7890A gas chromatograph system (Agilent Technologies, Palo Alto, CA, USA) equipped with an FID detector. The analytical column was an HP-5 (5%-phenyl)-methylpolysiloxane column (30 m × 0.32 mm i.d., 0.25 μm film thickness).

### 2.3. Experimental Animals

A total of 50 male Wistar rats, acquired from the Centre for Animal Experimentation (CECA) of the University of Malaga, were used. The initial weight of the animals was 200–220 g, and they were housed at the CECA for a quarantine period of seven days. After this time, the animals were identified and housed individually for the entire duration of this study. The diabetic animal must be maintained under ideal housing and hygienic conditions to avoid any unnecessary problems or suffering. Water intake (unrestricted) and feed intake (unrestricted) were always monitored, to assess whether changes in dietary habits related to the diabetic condition or the administration of the oils under study occurred.

### 2.4. Experimental Groups

The animals in this study were randomly distributed into the following experimental groups (10 male rats per group):

- Healthy non-diabetic rats (NDRs) (10 male rats). They served as controls for the variables determined under normoglycemic conditions. The procedures for administering any types of substances differed from the other groups only in that in this case, physiological saline was administered as a placebo.
- Diabetic control rats (DRs) (10 male rats). These animals were induced with experimental diabetes (see below), none of the study oils were administered, only insulin, with the aim of reducing mortality due to excessively high hyperglycemia.
- Treated diabetic rats. Once the presence of diabetes had been verified in each animal, they were given the oils under study:
- Extra-virgin olive oil (EVOO), at a dose of 0.5 mL/kg/day, by orogastric cannulation, for two months.
- Olive seed oil (OSO), at a dose of 0.5 mL/kg/day, by orogastric cannulation, for two months (10 male rats).
- Destoned and dehydrated olive oil (DDOO) at a dose of 0.5 mL/kg/day by orogastric cannulation for two months (10 male rats).

Each of the oils was administered once a day, always at the same time (8:00 a.m.), for 2 months, including the day of the end of the follow-up and the collection of the biological samples. We chose administration by orogastric cannula to ensure that 100% of the dose reached the stomach, thus avoiding a possible high variability in the absorption and blood absorption rates of the constituent components of the oils used. A soft cannula was used, without edges that could damage the esophageal wall, and the sample was deposited in the gastric cavity; the administration was always performed by the same person, who had ample experience in this administration technique.

The administered dose of the oils was chosen based on that used in human studies with EVOO (40–50 mL/day), i.e., approximately 0.5 mL/kg/day in people of 75–80 kg body weight [17,18].

According to the protocol, approved by the Experimentation Ethics Committee of the University of Malaga (Ref. CEUMA31-2018-A) and the Consejería de Agricultura, Ganadería, Pesca y Desarrollo Sostenible, Junta de Andalucía (Ref. 9/07/2019/124), the following signs and symptoms were checked daily in all the experimental animal:

- Presence of dyspnea, hemorrhage, stupor or cachexia (endpoint criteria).
- Presence of abnormal or increased secretions (no = 0 points; yes = 1 point); isolation or aggressive attitude towards conspecifics and/or investigator (no = 0 points; yes = 1 point); diarrhea (no = 0 points; yes = 1 point). In case of reaching 2 points, the end point criterion would have been applied.

No animals died during the experiment, and no end point criteria had to be applied.

### 2.5. Induction of Experimental Diabetes Mellitus

Diabetes mellitus was induced by the administration of streptozotocin (40 mg/kg) in a single intraperitoneal dose. Streptozotocin is an antibiotic that causes β-cell destruction in pancreatic islets and is used experimentally to produce a model of type 1 diabetes mellitus [19].

An animal was classified as diabetic if its blood glucose, measured with a FreeStyle glucose meter (Laboratorios Abbot S.A., Madrid, Spain) using blood from the tail vein, was 200 mg/dL for two consecutive days. To reduce the mortality of diabetic animals due to too high blood glucose levels, 4–6 IU/day of a long-acting insulin analogue—insulin detemir (Levemir^®^, Novo Nordisk A/S, Bagsværd, Denmark)—was administered subcutaneously. The aim of this study is to establish a state that would be equivalent to “the poor control of diabetes mellitus” in humans. To do this, it was necessary to maintain high blood glucose levels that would promote vascular alterations, but not high enough to cause the death of the animals. In our experience, insulin detemir manages to maintain these high blood glucose levels using a single dose per day. The dose was calculated according to the blood glucose of each animal: 2 IU if it was in the range 250–290 mg/dL; 4 IU if it was in the range 300–375; 6 IU if it was greater than 400 mg/dL; and only occasionally 8 IU if it exceeded 500 mg/dL.

### 2.6. Samples Collection

At the end of the two-month follow-up period, urine samples were obtained and collected for 24 h in metabolic cages (Tecniplast S.p.A., Buguggiate, Italy). After centrifugation at 3500× *g* for 10 min at 4 °C, the samples were divided into aliquots and frozen at −80 °C.

Subsequently, the animals, which had been fasting overnight, were anaesthetized with sodium pentobarbital intraperitoneally (40 mg/kg), and their blood was collected by puncture of the bifurcation of the iliac arteries. The animals were then decapitated.

The following biological samples were obtained from each animal:

- Urine, as described in the previous paragraph.
- Blood. Part of the blood was collected in tubes with anticoagulant (sodium citrate 3.8%, ratio 1:10). Part of the blood sample was poured into tubes with resin, without anticoagulation, to form serum; the blood samples were centrifuged at 4000 rpm for 10 min, and the resulting serum was separated, aliquoted and frozen at −80 °C until the time of analytical determinations.
- A segment of the aorta was obtained 0.5 cm anterior to the bifurcation of the renal arteries.

### 2.7. Analytical Techniques

#### 2.7.1. Biochemical Profile

All biochemical parameters were analyzed with an Atellica^®^CH autoanalyzer from Siemens Healthineers (Erlangen, Germany). All variables were determined according to the instructions of the kits used in the autoanalyzer. These determinations were as follows: glucose, total proteins, albumin, creatinine, total cholesterol, LDL cholesterol, HDL cholesterol and triglycerides.

#### 2.7.2. Early Variables of Vasculopathy

- Serum-oxidized low-density lipoprotein (oxLDL), a molecule that is oxidized by free radicals in the early stages of diabetic vasculopathy. It was determined by commercial ELISA, following the manufacturer’s instructions.
- Myeloperoxidase (MPOx), as a leukocyte activation index. It was determined by commercial ELISA, following the manufacturer’s instructions.
- The vascular adhesion molecule VCAM-1 as a biomarker of endothelial activation in the initial situation of vascular inflammation. It was determined by commercial ELISA, following the manufacturer’s instructions.

#### 2.7.3. Oxidative and Nitrosative Stress Variables

- Lipid peroxidation was measured through the determination of reaction products with thiobarbituric acid (TBARS), whose main representative is malondialdehyde (MDA). A commercial colorimetric kit with detection at 532 nm was used, following the manufacturer’s instructions.
- Global production of oxidative compounds, quantified through the determination of urinary 8-isoprostanes, compounds derived from the interaction of free radicals with arachidonic acid, producing a peroxidation of this fatty acid, which forms 8-epi-PGF_2α_ (8-isoprostanes) without any enzymatic intervention. It was determined by commercial ELISA, following the manufacturer’s instructions.
- DNA damage caused by free radicals, measured through the determination of 8-hydroxy-2-deoxyguanosine. Determined by commercial ELISA, following the manufacturer’s instructions.
- Peroxynitrite production, to assess nitrosative stress, i.e., the formation of free radicals derived from nitric oxide (NO). These radicals nitrate the amino acid tyrosine in a 1:1 ratio, forming 3-nitrotyrosine. It was determined by commercial ELISA, following the manufacturer’s instructions.
- Total antioxidant capacity (TAC) as an index of the capacity of a sample to exert an antioxidant defense using all its free radical inhibition mechanisms. The TAC assay is based on the reduction of Cu^++^ to Cu^+^ by antioxidants such as uric acid and the reaction with a chromogen, determining the absorbance at 490 nm, using a commercial colorimetric kit.
- Concentration of reduced glutathione (GSH), the main tripeptide used by the body as a storehouse of a quantitatively important antioxidant system. A commercial colorimetric test was used, whose instructions were followed to obtain GSH concentrations.
- Glutathione peroxidase activity (GSHpx), an enzyme that oxidizes GSH to GSSG, consuming NADPH, which interacts with free radicals and decreases their oxidative capacity. A commercial colorimetric test based on a spectrophotometric kinetic method was used.

#### 2.7.4. Thrombogenic Related Variables

- Platelet aggregometry. The ability of platelets to aggregate was quantified using a whole-blood electrical impedance aggregometer (Chrono-Log 590, Chrono-Log Corp., Haverton, PA, USA), using collagen (10 µg/mL) as an inducer of platelet aggregation. Maximum platelet aggregation intensity (Imax, ohms) was quantified 10 min after addition of collagen.
- Thromboxane production. The presence of a stable metabolite of thromboxane A_2_, 11-dehydro-thromboxane B_2_, a product of the overall formation of this prostanoid in the whole organism, was detected in urine. It was determined by commercial ELISA, following the manufacturer’s instructions.
- Prostacyclin production. The presence of a stable metabolite of prostacyclin, 6-keto-prostaglandin F_1α_, a product of the overall formation of this prostanoid in the whole organism, was detected in urine. It was determined by commercial ELISA, following the manufacturer’s instructions.

#### 2.7.5. Vascular Morphometric Evaluation

The morphometric study was performed on the aortic segment (see Samples Collection). The vascular segment was fixed with 10% paraformaldehyde for 48 h using the standard paraffin-embedding method. We obtained 5 mm sections and stained them with hematoxylin and eosin.

The samples were examined under a digitized light microscope. Morphometric analysis was performed using Visilog v. 6.3 software licensed by the Central Research Support Service of the University of Malaga (SCAI).

From each arterial sample, 10 randomly selected sections of 5 to 7 slides were analyzed. In each section, we quantified the variables, lumen area (LA) and area of the whole arterial section (WA). Arterial wall area (AWA) was calculated as follows:

AWA = WA − LA

In addition, stained aortic sections were used to count the number of smooth muscle cell nuclei in the tunica media. The image was segmented into a new binary image, with black representing the nuclei. The number of cell nuclei was calculated within four fields of approximately 10,000 µm^2^ at 0°, 90°, 180° and 270° in each section.

### 2.8. Statistical Analysis

Data in the text, tables and figures are expressed as mean ± standard deviation (SD) of 10 animals. All statistical analyses were performed with the Statistical Package for Social Sciences v. 25.0 (SPSS Co., Chicago, IL, USA). One-way analyses of variance, followed by Bonferroni transformation and Student’s *t*-tests for unpaired data, were used. In all cases, statistical significance was assumed at a *p*-value < 0.05.

## 3. Results

The zoometric variables were altered in diabetic control animals (Table 2), in accordance with the basic symptomatology of diabetes: lower increase in body weight (20% lower with respect to healthy controls), higher food (36% more) and drink intake (2.7 times higher) and diuresis (2.2 times higher). Although a tendency to reduce these alterations was observed after the administration of the three oils studied, only polyuria values reached statistical significance (Table 2).

Blood glucose was statistically higher in diabetic control animals (4.2 times higher) (Table 3); the oils used did not modify these values statistically after two months of treatment.

Diabetic animals showed a significantly higher serum concentration of total cholesterol, LDL cholesterol and triglycerides, than non-diabetic animals (36.3%, 98.3% and 2.4 times higher, respectively) (Table 3). All three types of oils reduced LDL cholesterol and triglyceride values and increased HDL cholesterol values; total cholesterol levels were reduced after treatment with EVOO and DDOO, but OSO did not significantly modify them.

Regarding cardiovascular variables related to diabetic vasculopathy (Table 4), these increased in diabetic control animals: 3.7 times the serum concentration of myeloperoxidase, 1.6 times VCAM-1 and 1.8 times oxidized LDL. The administration of the three oils used reduced the concentration of these biomarkers, except for VCAM-1 in diabetic animals treated with OSO.

**Figure 2:**
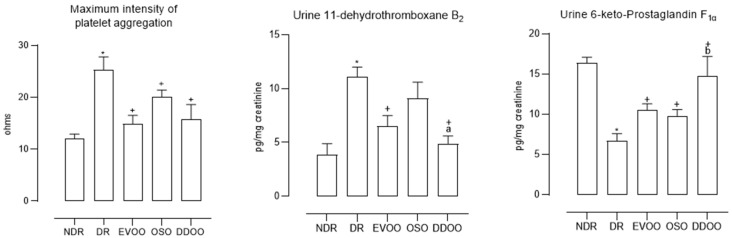
Maximum intensity of whole blood platelet aggregation induced with collagen, and urine concentration of 11-dehydro-thromboxane B_2_ and 6-keto-prostaglandin F_1α_ from non-diabetic rats (NDR), diabetic control rats (DR) and diabetic animals treated with 0.5 mL/kg body weight of extra-virgin olive oil (EVOO), olive seed oil (OSO) or destoned and dehydrated olive oil (DDOO). * *p* < 0.05 with respect to NDR. ^+^ *p* < 0.05 with respect to DR. ^a^ *p* < 0.05 with respect to OSO. ^b^ *p* < 0.05 with respect to EVOO and OSO. *n* = 10 rats per group.

Platelet activation was 2.1 times higher in diabetic control animals (Figure 2), being reduced by the three oils, the effect of EVOO and OSO being greater than that of DDOO, but these differences did not reach statistical significance. Thromboxane production was 2.8 times higher in diabetic controls than in normoglycemic animals and was reduced by the three oils, although the effect of DDOO was significantly greater (Figure 2). The prostacyclin production was 60.0% lower in diabetic animals compared to non-diabetic animals, increasing after the administration of the three types of oils, although as we observed with the production of thromboxane, DDOO produced the greatest effect in relation to EVOO and OSO (Figure 2).

With respect to oxidative and nitrosative stress variables (Table 5), diabetic animals showed higher values of oxidative parameters and lower values of antioxidant parameters, compared to normoglycemic animals. In diabetic animals, 2.1 times higher concentrations of TBARS, 1.6 times those of 8-hydroxy-2-deoxyguanosine, 7.3 times those of urinary F2-isoprostanes and 4.3 times those of 3-nitrotyrosine were determined. Regarding antioxidant variables, diabetic animals showed a 25.6% reduction in total antioxidant capacity, 27.8% in serum GSH concentration and 72.1% in glutathione peroxidase activity. The administration of the three oils used significantly modified the oxidative stress profile, mainly by reducing the oxidative pathways and lowering the amount of antioxidant defense (Table 5). DDOO administration showed a greater effect than OSO and EVOO in inhibiting TBARS and 8-hydroxy-2-deoxyguanosine and in increasing GSH and glutathione peroxidase activity (Table 5).

**Figure 3:**
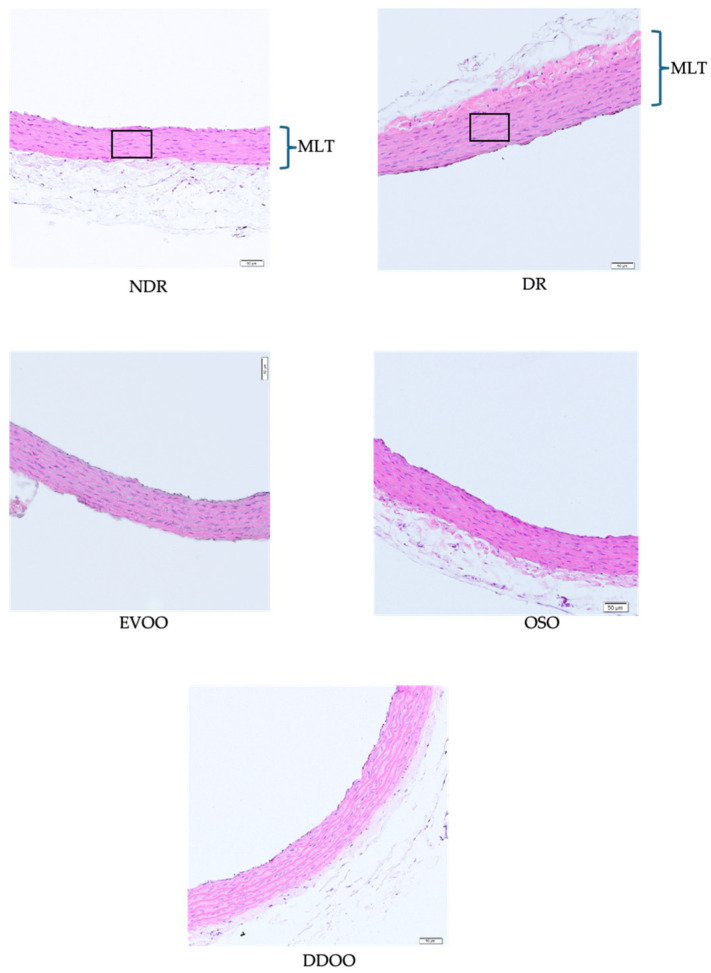
Representative images of aortic sections from non-diabetic rats (NDR), control diabetic rats (DR) and DR treated with 0.5 mL/kg body weight of extra-virgin olive oil (EVOO), olive seed oil (OSO) or destoned and dehydrated olive oil (DDOO). Hematoxylin-Eosin 20X. MLT: muscle layer thickness. Squares indicate areas of varying density of vascular smooth muscle fibers.

Figure 3 shows representative examples of aortic vascular wall sections in the different study groups, whose global morphometric quantification is shown in Table 6. Diabetic animals showed a significant increase in the arterial wall area (42.7% greater) and in cell density in the muscular layer (31.1% greater), in comparison with non-diabetic animals. The administration of the three oils reduced the arterial wall area (22.4%, 21.8% and 20.4% reduction with EVOO, OSO and DDOO respectively) and normalized muscle cell density (28.2%, 24.5% and 28.1% reduction with EVOO, OSO and DDOO respectively), with no statistical differences among the three types of oils.

## 4. Discussion

By establishing a new way to utilize olive by-products, the olive industry can reduce waste in producing EVOO. However, the oils under study must meet one main characteristic, in addition to confirming that they reduce waste and yield more products: they must produce at least the same beneficial effects on health as those already recognized in EVOO. In the production of DDOO, 3 kg of by-products are generated instead of 4 kg to produce one liter of DDOO oil, and by pitting, the OSO is obtained from the stone seed, generating animal feed, and stone fragments for use as fuel. This study demonstrates, for the first time, that both types of oils (OSO and DDOO) have the same qualitative profile of preventive effects on biomarkers of vasculopathy in diabetes mellitus, although some quantitative differences are observed with respect to EVOO.

In general terms, we can summarize the main results obtained in the two following sentences: (1) the oil obtained from the seeds of Picual variety olives (OSO) shows very similar effects to EVOO in most of the variables determined; (2) destoning and dehydration of the pulp of Picual variety olives (DDOO) shows a greater effect than EVOO and OSO on some variables related to vascular inflammation (VCAM-1), hemostatic imbalance (thromboxane and prostacyclin) and oxidative stress (lipid peroxides, 8-OH-2-deoxyguanosine, reduced glutathione and glutathione peroxidase activity).

The experimental model used reproduces almost all the modifications of the main cardiovascular biomarkers involved in the development of diabetic vasculopathy [20,21], which are significantly altered in experimental diabetic animals.

With respect to EVOO-treated animals as a reference treatment, in this same experimental method, it was demonstrated in previous studies that EVOO decreases a large part of the cardiovascular biomarkers altered by experimental diabetes, as well as some morphological lesions of diabetic microangiopathy [22,23].

It is now widely accepted that this preventive effect of EVOO is mainly due to its antioxidative and anti-inflammatory effects. These effects are also attributed to the polyphenolic components of EVOO, which have demonstrated a preventive effect in this same experimental model of diabetes, especially hydroxytyrosol, alone or in association with 3’,4’-dydhydroxyphenylglycol [7,10]. The results obtained with EVOO are due to a direct effect on the biochemical mechanisms involved in diabetic vasculopathy (mainly oxidative stress, vascular inflammation and endothelial and platelet dysfunction), rather than due to reduction in blood glucose levels since these are not significantly modified in animals treated with EVOO. In human studies, the relationship between EVOO and glycemic control refer to type 2 diabetes mellitus, in the sense of delaying the use of oral antidiabetic drugs [24]. The experimental model used in this study reproduces type 1 diabetes mellitus; hence, the changes in glycemia after ingestion of the oils are not significant.

The administration of OSO and DDOO to diabetic animals, always in comparison with EVOO, showed a beneficial effect on all biomarkers of each of the phases of the pathophysiology of diabetic vasculopathy, from the lipid profile, oxidative stress, thrombogenic variables and early phase biomarkers of vascular inflammation. A differential fact between EVOO and DDOO is the presence of the olive stone in the process of obtaining the final oil. Destoned olives have been shown to improve the olive-oil yield [25], as well as to increase the concentration of polyphenols in olive pulp [26]. This study demonstrates that the destoning process improves the effect profile of the oil on cardiovascular biomarkers in experimental diabetes, but also demonstrates that the oil obtained from the stone seed (OSO) has beneficial effects on these biomarkers.

It is widely accepted that EVOO, especially as the main source of fat in the Mediterranean diet, shows a preventive effect on cardiovascular events in patients with recognized risk factors, including diabetes mellitus [3]. Likewise, clinical and experimental studies have described an effect of EVOO on most of the mechanisms involved in the development of diabetic vasculopathy: control of the lipid profile [27], decrease of endothelial dysfunction [28] and of the main thrombogenic factors [29]. It is also accepted that in these effects, the antioxidative action of the polyphenolic components of EVOO plays a fundamental role [3,4]. From a qualitative point of view, OSO and DDOO share these cardiovascular prevention mechanisms with EVOO, so they should have an overall vasculopathy prevention action similar to that demonstrated with EVOO, including diabetic vasculopathy [3,4]. From a quantitative point of view, we cannot compare the effects of OSO and DDOO in the literature, since there are no comparative studies at this level, only the study by Sánchez-Rodriguez et al. [30] compares EVOO enriched with triterpenoid compounds with EVOO with the same amount of alcoholic polyphenols but less amount of triterpenoid compounds, demonstrating that the effects of OSO and DDOO can be compared.

Although we have not performed direct experiments, we could hypothesize about the responsibility of the components of these oils that could explain the quantitative differences in the effects between them. Basically, polyphenolic compounds and recently triterpenoids have been related to the cardiovascular protection of EVOO. When analyzing the concentration of these compounds in the three types of oils, we observed that the total amount of polyphenols does not differ greatly (703 mg/kg in EVOO, 530 mg/kg in OSO and 689 mg/kg in DDOO). The total concentration of polyphenols is in a range that can be defined as high [17], so it would clearly explain the effects found in the experimental model of diabetes mellitus used in this study [8,10], but it does not explain the difference between the three types of oils studied.

Other compounds that are related to the cardiovascular protective effect of EVOO are triterpenoids. In DDOO, the content of triterpenoids is clearly higher than in EVOO and OSO (913 mg/kg, compared to 113 mg/kg in OSO and 75 mg/kg in EVOO) (Table 1). These triterpenoid compounds could be responsible for the greater antioxidative effect of DDOO, and we could even postulate a possible synergistic effect with polyphenols, as has been previously demonstrated with hydroxytyrosol and oleocanthal [7].

The most important differences found in this study focus on the antioxidative effect of DDOO with respect to OSO and EVOO. The polyphenol content could explain its antioxidative effect [24] but not the difference with the other two oils. In this sense, the main difference of DDOO lies in the concentration of triterpenoid derivatives (oleanolic acid, maslinic acid and ursolic acid). A study in healthy volunteers has shown that the administration of an extra-virgin olive oil enriched with triterpenoid compounds (oleanoic acid and maslinic acid) and equal total polyphenol content to extra-virgin olive oil shows a greater inhibitory effect on urinary excretion of 8-isoprostanes and 8-hydroxy-2-deoxyguanosine [30]. In the present study, we found that the administration of DDOO (with a higher content of triterpenoid compounds and a similar content of total polyphenols) shows a greater overall antioxidative effect than the other two types of oils. The antioxidative effects of oleanolic acid [31,32], maslinic acid [33,34] and ursolic acid [35], are known, relating this effect to an anti-inflammatory effect and a cytoprotective effect at the cardiac or cerebral level.

Other variables that are affected to a greater extent by DDOO are those related to thromboxane and prostacyclin production. In this regard, it has been demonstrated that oleanonic acid induces the production and release of prostacyclin in vascular smooth muscle cell cultures through a mechanism related to type 2 cyclooxygenase [36]; also, maslinic acid is known to exert an antiplatelet effect induced by activation of the intraplatelet thromboxane pathway [37], and plantain extracts (rich in ursolic acid) were shown to inhibit thromboxane A_2_ production in inflammation models [38]. The negative effect of oxidative stress on prostacyclin production could be inhibited by the oils used, thus explaining the smaller decrease in prostacyclin in treated diabetic animals (Figure 2), in direct proportion to the demonstrated antioxidative effect (Table 5).

To summarize all the results obtained with the oils used, Table 7 shows the percentages of change in each group with respect to the diabetic control animals, considering the mean of the changes obtained by groups of variables and the percentages of change regardless of whether they are increases or decreases (see the Results section). This table shows the greater percentage effect of DDOO, with respect to EVOO and OSO, on oxidative stress and prostanoid production.

Establishing the mechanism by which DDOO shows a greater effect on cardiovascular biomarkers in the experimental model of type 1 diabetes mellitus requires future studies that analyze the molecular mechanism by which alcoholic polyphenols and triterpenoids can modify these biomarkers and exert their influence on cardiovascular function. However, the use of these oils, especially DDOO, has served to draw attention to the role of triterpenoids in the development of diabetic vasculopathy. It has been previously described that alcoholic polyphenols have been shown to play an important regulatory role in the biochemical mechanisms that condition the vascular complications of diabetes [8,9,10,12,14,39] and that some of the triterpenoids exert an effect on some of these mechanisms [31,32,33,34,36,37,38]. However, the main differences observed in this study have been at the level of its antioxidative effects, which is why we could hypothesize that a greater decrease in the oxidative stress of DDOO could explain, at least in part, the greater effect of this oil on cardiovascular biomarkers in diabetes mellitus. No studies have yet been published on OSO and DDOO in relation to these mechanisms, so any explanations would be hypothetical. Specific biochemical and molecular studies are needed to analyze the possible effect of triterpenoids, alone or in association with alcoholic polyphenols, on each of the biochemical pathways involved in diabetic vasculopathy.

This study has several limitations, including the following: (1) we have made an approximation of the effects of these oils on cardiovascular and oxidative stress biomarkers, with the aim of assessing in proportional terms the effect between them, but we have not carried out mechanistic experiments, analyzing the specific role of each of the phenolic compounds found in these oils, as well as their possible potentiation at the concentrations at which they have been determined in EVOO, OSO and DDOO; (2) kinetic studies were not carried out to determine how much of these compounds reach the blood and their organic metabolism. Future studies on the effect of triterpenoids, alone or in association with alcoholic polyphenols, on these biomarkers are required, and these experiments are already underway in our laboratory.

## 5. Conclusions

In conclusion, this study shows that two oils obtained from olive derivatives have at least similar effects to those of EVOO, even the oil obtained from destoned and dehydrated olives, with a high content of triterpenoid derivatives, shows an even greater antioxidative effect and modification in prostanoid production than EVOO.

## Figures and Tables

**Table 1:** Composition of extra-virgin olive oil (EVOO), olive seed oil (OSO) and destoned and dehydrated olive oil (DDOO).

	EVOO	OSO	DDOO
Acidity (%)	0.12	37.26	0.12
Peroxide value (mEqO_2_/kg)	8.2	3.1	12.9
K270	0.16	1.39	0.19
K232	1.88	3.23	1.47
Delta K	<0.01	0.02	<0.01
Ethyl oleate (mg/kg)	7	4506	5
Waxes (mg/kg)	34	335	59
Fatty acid composition			
Myristic acid (%)	0.01	0.04	0.01
Palmitic acid (%)	12.24	9.86	13.62
Palmitoleic acid (%)	1.10	0.26	1.22
Margaric acid (%)	0.05	0.09	0.05
Margaroleic acid (%)	0.08	0.07	0.07
Stearic Acid (%)	3.47	2.87	2.62
Oleic Acid (%)	76.60	68.52	77.47
Linoleic Acid (%)	4.95	16.34	3.29
Linolenic Acid (%)	0.71	0.29	0.82
Arachidic Acid (%)	0.39	0.56	0.40
Eicosanoic Acid (%)	0.20	0.55	0.22
Bhenenic Acid (%)	0.10	0.30	0.11
Lignoceric acid (%)	0.05	0.15	0.07
Total sterols (mg/kg)	1367	2653	1528
Brassicasterol (%)	<0.1	<0.1	<0.1
Cholesterol (%)	3.1	5.0	2.9
Stigmasterol (%)	0.6	1.8	1.1
B-Sitosterol (%)	94.6	90.1	94.9
D7-Stigmastenol (%)	0.3	1.6	0.3
Erythrodiol + Uvaol (%)	1.0	1.1	3.6
Total triterpenic acids (mg/kg)	75.40	113.64	913.74
Oleanolic acid (mg/kg)	22.26	41.02	402.35
Maslinic acid (mg/kg)	53.14	72.62	498.14
Ursolic acid (mg/kg)	<5.00	<5.00	13.25
Chlorophyll pigments (mg/kg)	23.63	8.16	26.25
Carotenoid pigments (mg/kg)	7.69	9.33	8.12
Squalane (mg/100 g)	440	9	664
Tocoferoles (mg/kg)	342	11	394
Total phenols (mg/kg)	703.48 ± 11.42	530.04 ± 5.63	689.39 ± 39.43
3,4-dihydroxyphenylglycol	1.07	0.215	-
Hydroxytyrosol	5.825	29.8	8.88
Tyrosol	1.9	22.755	14.04
Vanillin	-	-	-
Vanillic acid	-	3.13	-
Hydroxytyrosol acetate	-	-	26.24
Nuzhenide	-	14.26	4.46
Oleuropein derivative 1	-	26.76	-
Oleuropein derivative 2	36.52	23.82	22.66
Ligustroside derivative	49.46	-	49.42
Sum	94.775	120.74	125.7
Hydroxytyrosol (HT) potential (ppm)	25	45	30
% of potential HT approx.	0.0025	0.0045	0.0030

**Table 2:** Mean values (mean ± standard deviation) corresponding to the zoomometric parameters of non-diabetic rats (NDR), diabetic control rats (DR) and diabetic animals treated with 0.5 mL/kg body weight of extra-virgin olive oil (EVOO), olive seed oil (OSO) or destoned and dehydrated olive oil (DDOO).

	NDR	DR	EVOO	OSO	DDOO
*n*	10	10	10	10	10
Body weight (g)					
Day 1	239 ± 5.1	235 ± 4.7	233 ± 5.5	235 ± 5.3	238 ± 6.0
Day 60	380 ± 6.1	343 ± 16.0 *	336 ± 30.1	317 ± 7.9	335 ± 30.0
% increase	59.2 ± 10.1	39.0 ± 15.4 *	43.3 ± 19.2	38.1 ± 16.4	47.5 ± 17.0
Food ingested (g/day)	20.7 ± 2.1	28.3 ± 4.4 *	21.3 ± 2.9	22.4 ± 0.8	24.3 ± 4.6
Drink ingested (mL/day)	37.9 ± 14.4	105 ± 45.0 *	80.8 ± 22.8	75.5 ± 19.5	80.8 ± 15.2
Diuresis (mL/day)	15.6 ± 1.3	34.3 ± 3.2 *	17.1 ± 1.9 ^+^	15.9 ± 2.6 ^+^	17.9 ± 11.7 ^+^

* *p* < 0.05 with respect to NDR; ^+^ *p* < 0.05 with respect to DR.

**Table 3:** Mean values (mean ± standard deviation) corresponding to the overall serum analytical variables and lipid profile of non-diabetic control rats (NDR), diabetic controls rats (DR) and diabetic animals treated with 0.5 mL/kg body weight of extra-virgin olive oil (EVOO), olive seed oil (OSO) or destoned and dehydrated olive oil (DDOO).

	NDR	DR	EVOO	OSO	DDOO
*n*	10	10	10	10	10
Blood glucose (mg/dL)	86.4 ± 5.2	452 ± 9.4 *	538 ± 103	499 ± 99.0	481 ± 104
Serum creatinine (mg/dL)	0.3 ± 0.01	0.7 ± 0.03 *	0.6 ± 0.06 ^+^	0.6 ± 0.07 ^+^	0.5 ± 0.04 ^+^
Total proteins (g/dL)	5.5 ± 0.1	5.3 ± 0.2	5.5 ± 0.3	5.2 ± 0.2	5.1 ± 0.2
Albumin (g/dL)	1.5 ± 0.06	1.4 ± 0.2	1.3 ± 0.1	1.4 ± 0.08	1.3 ± 0.06
Total cholesterol (mg/dL)	55.5 ± 8.9	75.6 ± 3.6 *	63.0 ± 1.9 ^+^	73.3 ± 2.6	60.9 ± 5.7 ^+^
LDL cholesterol (mg/dL)	18.7 ± 1.2	37.1 ± 4.8 *	21.6 ± 2.1 ^+^	24.3 ± 2.3 ^+^	22.7 ± 1.3 ^+^
HDL cholesterol (mg/dL)	18.9 ± 3.9	17.7 ± 1.6	24.9 ± 2.0 ^+^	25.9 ± 1.7 ^+^	24.3 ± 3.3 ^+^
Triglycerides (mg/dL)	53.9 ± 11.5	130 ± 6.9 *	95.5 ± 3.1 ^+^	103 ± 5.7 ^+^	80.8 ± 5.3 ^+,a^

* *p* < 0.05 with respect to NDR; ^+^ *p* < 0.05 with respect to DR; ^a^ *p* < 0.05 with respect to OSO. HDL: high-density lipoproteins; LDL: low-density lipoproteins.

**Table 4:** Mean values (mean ± standard deviation) corresponding to variables of vascular and platelet biochemistry of non-diabetic control rats (NDR), diabetic controls rats (DR) and diabetic animals treated with 0.5 mL/kg body weight of extra-virgin olive oil (EVOO), olive seed oil (OSO) or destoned and dehydrated olive oil (DDOO).

	NDR	DR	EVOO	OSO	DDOO
*n*	10	10	10	10	10
MPOx (ng/mL)	0.7 ± 0.07	2.7 ± 0.2 *	1.6 ± 0.5 ^+^	1.9 ± 0.1 ^+^	1.8 ± 0.1 ^+^
VCAM-1 (ng/mL)	4.8 ± 0.7	7.6 ± 0.7 *	4.7 ± 1.3 ^+,a^	7.2 ± 0.4	5.2 ± 1.1 ^+,a^
oxLDL (ng/mL)	140 ± 20.2	257 ± 10.5 *	214 ± 11.9 ^+^	227 ± 10.7 ^+^	202 ± 18.3 ^+^

* *p* < 0.05 respect to NDR; ^+^ *p* < 0.05 respect to DR; ^a^ *p* < 0.05 respect to OSO. MPOx: myeloperoxidase; oxLDL: oxidized low-density lipoprotein; VCAM-1: type 1 vascular cyto-adhesive.

**Table 5:** Mean values (mean ± standard deviation) corresponding to serum oxidative and nitrosative stress variables of non-diabetic control rats (NDR), diabetic controls (DR) and diabetic animals treated with 0.5 mL/kg body weight of extra-virgin olive oil (EVOO), olive seed oil (OSO) or destoned and dehydrated olive oil (DDOO). * *p* < 0.05 respect to NDR; ^+^ *p* < 0.05 respect to DR; ^a^ *p* < 0.05 respect to OSO; ^b^ *p* < 0.05 respect to OSO and EVOO. TBARS: thiobarbituric acid reactive substances (lipid peroxides); 8-OH-dG: 8-hydroxy-2-deoxyguanosine; TAC: total antioxidant capacity; GSH: reduced glutathione; GSHpx: glutathione peroxidase activity.

	NDR	DR	EVOO	OSO	DDOO
*n*	10	10	10	10	10
TBARS (nmol/mg prot)	4.0 ± 0.8	8.6 ± 0.7 *	3.7 ± 0.9 ^+^	4.9 ± 0.5 ^+^	1.7 ± 0.3 ^+,b^
8-OH-dG (ng/mL)	15.5 ± 0.4	25.3 ± 1.6 *	3.6 ± 0.8 ^+,a^	6.3 ± 0.9 ^+^	1.3 ± 0.2 ^+,a^
F_2_-isoprostanes (ng/mg creatinine)	6.4 ± 0.5	47.1 ± 0.6 *	14.0 ± 0.8 ^+^	13.4 ± 0.5 ^+^	14.8 ± 0.8 ^+^
3-nitrotirosine (pg/mL)	14.2 ± 0.9	61.9 ± 3.4 *	43.8 ± 1.2 ^+^	44.2 ± 0.8 ^+^	39.8 ± 2.2 ^+^
TAC (U/mL)	17.1 ± 0.5	12.7 ± 0.7 *	14.9 ± 1.6 ^+^	15.7 ± 1.5 ^+^	15.6 ± 0.5 ^+^
GSH (nmol/mL)	121 ± 7.5	87.7 ± 6.7 *	94.8 ± 3.1	92.1 ± 3.1	123 ± 2.0 ^+,b^
GSHpx (nmol/min/mL)	26.8 ± 1.0	7.5 ± 1.2 *	17.0 ± 3.0 ^+^	15.9 ± 2.6 ^+^	24.7 ± 2.2 ^+,b^

**Table 6:** Mean values (mean ± standard deviation) corresponding to aortic morphological variables of non-diabetic control rats (NDR), diabetic control rats (DR) and diabetic animals treated with 0.5 mL/kg body weight of extra-virgin olive oil (EVOO), olive seed oil (OSO) or destoned and dehydrated olive oil (DDOO).

	NDR	DR	EVOO	OSO	DDOO
*n*	10	10	10	10	10
Arterial wall area (µm^2^)	104 ± 5.6	144 ± 3.0 *	114 ± 4.1 ^+^	115 ± 5.5 ^+^	117 ± 10.1 ^+^
Number of muscular cells (*n* × 10^5^/µm^2^)	40.3 ± 2.1	52.9 ± 2.7 *	38.7 ± 0.9 ^+^	40.7 ± 1.3 ^+^	38.7 ± 1.6 ^+^

* *p* < 0.05 respect to NDR; ^+^ *p* < 0.05 respect to DR.

**Table 7:** Percentages of change in the different groups of variables analyzed, considering the average variation of each of the parameters in each group of diabetic animals treated with extra-virgin olive oil (EVOO), olive seed oil (OSO) or destoned and dehydrated olive oil (DDOO), with respect to control diabetic rats.

	EVOO	OSO	DDOO
Lipid profile (a)	31.8	29.6	24.7
Early vascular inflammation biomarkers (b)	33.0	17.1	30.2
Oxidative stress (c)	44.3	41.1	64.7
Prostanoids (d)	45.7	29.0	70.7
Morphology (e)	25.2	23.2	24.2

(a) Total cholesterol, cholesterol-LDL, cholesterol-HDL and triglycerides. (b) Myeloperoxidase, VCAM-1 and oxidized LDL. (c) Oxidative and antioxidative parameters. (d) 11-dehydrothromboxane B_2_ and 6-keto-prostaglandin F_1α_. (e) Arterial wall area and number of muscular cells.

## Data Availability

The data presented in this study are available in this article.
